# Access to specialized outpatiecare: an analysis of regulation in Brazilian settings

**DOI:** 10.11606/s1518-8787.2026060007411

**Published:** 2026-06-15

**Authors:** Thais Barros Zanette da Silva, Jozinélio Severino Teixeira, Carlos Amilcar Salgado, Luciano de Paula Camilo, Claudilene Sousa Fortaleza, Fábio Ferreira Amorim, Leila Bernarda Donato Göttems

**Affiliations:** I Universidade do Distrito Federal. Escola Superior de Ciências da Saúde. Brasília, DF, Brasil; II Universidade de Brasília. Faculdade de Ciências da Saúde. Brasília, DF, Brasil; III Organização Pan-Americana de Saúde. Brasília, DF, Brasil

**Keywords:** Unified Health System, Health Care Coordination and Monitoring, Health Services Accessibility, Secondary Health Care, Health Policy, Planning and Management, Telemedicine

## Abstract

**OBJECTIVE:**

To analyze how different subnational governments, based on common national guidelines, have developed their own models for regulating access to specialized care, with varying degrees of innovation, institutionalization, and integration of telehealth.

**METHOD:**

A descriptive and exploratory study with a qualitative approach that comparatively analyzes the implementation of regulations governing access to specialized care. Data were collected through document analysis, literature review, and, in some cases, interviews with key stakeholders, consolidated into detailed descriptions of each scenario to form the research *corpus*. Analysis was conducted using the Consolidated Framework for Implementation Research (CFIR). Experiences were selected from the municipalities of Curitiba (PR), Belo Horizonte (MG), Porto Alegre (RS), and São Paulo (SP), as well as the states of Ceará, Santa Catarina, Rio Grande do Sul, and the Federal District.

**RESULTS:**

The eight Brazilian experiences with outpatient regulation are characterized by diversity in organizational models, degrees of institutionalization of teleconsultation, and integration across levels of care. The adoption of technologies, access regulation protocols, and computerized systems varied across locations, influenced by structural, regulatory, and contextual factors. When integrated, teleconsultation improved the quality of referrals and increased problem-solving capacity in primary care, but its implementation remains uneven across the country.

**CONCLUSION:**

The implementation of access regulation to specialized care in the Unified Health System varied according to institutional, organizational, and technological capacities. Weaknesses were identified in the integration of health information systems in the organization and delivery of specialized care, with structural inequalities; in governance, with low standardization of protocols; limited integration between Primary Health Care and specialized care; and professional resistance to change. Regions with better outcomes integrated teleconsultation into outpatient regulation; clinical and access protocols adopted health information systems integrated with analytical intelligence resources and institutionalized regulatory guidelines. The CFIR highlighted the influence of contexts, actors, and processes, reinforcing the importance of national guidelines for improving local organizations.

## INTRODUCTION

The federative and decentralized structure of Brazil’s Sistema Único de Saúde (SUS - Unified Health System) has facilitated expanded access to Primary Health Care (PHC), but it still perpetuates the fragmentation of Redes de Atenção à Saúde (RAS - Health Care Networks ) and inequality in the provision of specialized care across the country’s regions^1^ , especially in small municipalities, due to technical, operational, and financial constraints^2^ . Among the strategies to address this scenario, the Política Nacional de Regulação em Saúde (PNR - National Health Regulation Policy)^3^ stands out, promoting intergovernmental cooperation, resource sharing, and integration across levels of care. Published in 2008 and revised in 2025, the PNR has a regulatory framework, operational structure, financing, and information system aimed at organizing care flows, mediating the relationship between supply and demand, and promoting the rational use of available resources^4^. Its implementation, however, reveals significant heterogeneity in the Brazilian context, due to the federal nature of the system, the coexistence with the private sector, and inequalities in institutional capacity among federal entities^5^.

In Brazil, health regulation is organized into three interdependent dimensions: access, care, and health systems^6^. Access regulation plays an essential role in the coordination between PHC and specialized care, through regulation centers, protocols, and computerized systems^7,8^. The concept has evolved in official documents (Figure) as a set of actions aimed at organizing and controlling access to specialized care, based on criteria of risk, vulnerability, health needs, and resource availability^9,10^.

**Figure:**
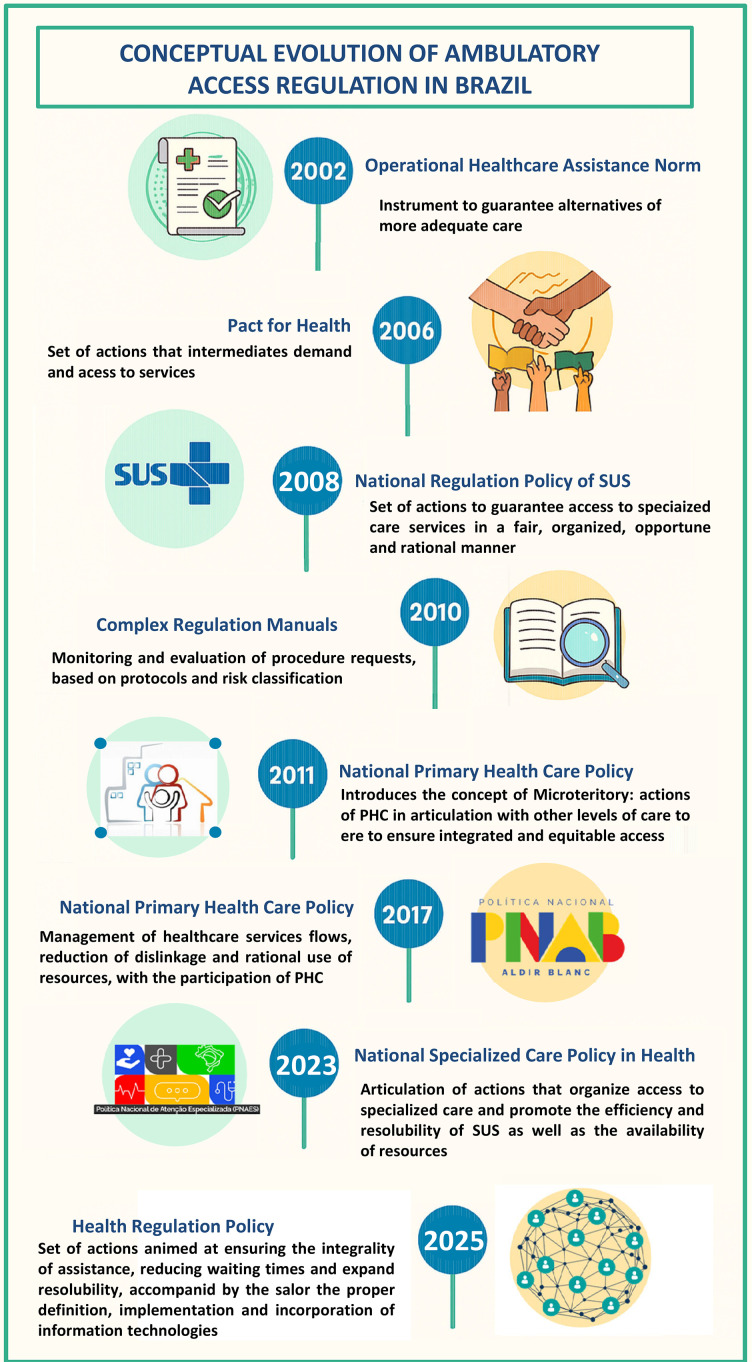
Infographic showing the conceptual evolution of Outpatient Access Regulation in Brazil.

Source: prepared based on references 9,10,21,22.

Health regulation has gained significant visibility and strength since mid-2010 through the National Telehealth Brazil Networks Program, by qualifying referrals and optimizing the use of outpatient care services^11^. New modes of interaction were created, notably teleconsultation, defined as a consultation between health professionals to clarify clinical questions, conducted via bidirectional telecommunication, whether synchronous or asynchronous. Evidence indicates that this practice has been effective in reducing waiting lists, expanding the problem-solving capacity of PHC, and improving the quality of clinical decisions^14,15^. However, its implementation has varied according to contextual, political, and institutional factors^16^.

This study aims to analyze how different subnational governments, based on common national guidelines, have developed their own models for regulating access to emergency care, with varying degrees of innovation, institutionalization, and integration of telehealth.

## METHODS

### Study Design

A descriptive and exploratory study with a qualitative approach that comparatively analyzes the implementation of regulations governing access to primary care in three states—Ceará (CE), Santa Catarina (SC), and Rio Grande do Sul (RS); four municipalities—Belo Horizonte-MG (BH), Porto Alegre-RS (POA), São Paulo-SP (SPO), Curitiba-PR (CUT)—and the Federal District (DF), organized politically and administratively as a state and municipality.

The selection of settings sought to represent different contexts and institutional arrangements, based on criteria of institutional relevance, availability of public information, and national recognition, enabling the comparison of distinct models of governance and integration between PHC and specialized care. The settings exhibit demographic, socioeconomic, and organizational heterogeneity (Table), share autonomy in the management of the SUS, and face common challenges related to access to specialized care.

**Table:** Epidemiological and demographic indicators of the selected scenarios.

Scenario	Population (number of inhabitants)	HDI	Life expectancy at birth in years	Infant mortality per 1,000 live births	PHC coverage
DF	2,982,818	0.814	79.7	8.5	76.8%
SC	7,338,473	0.774	77.4	9.80	88.7%
CE	9,240,580	0.682	79.01	11.9	77.2%
RS	10,880,506	0.771	75.38	9.59	68.0%
BH	2,315,560	0.797	78.81	8.19	81.3%
SPO	11,451,245	0.783	82.62	10.23	70.6%
CUT	1,773,718	0.823	78.5	7.1	75.8%
POA	1,332,570	0.805	77.45	7.78	61.0%

HDI: Human Development Index; PHC: Primary Health Care; DF: Federal District; SC: Santa Catarina; CE: Ceará; RS: Rio Grande do Sul; BH: Belo Horizonte (MG); SPO: São Paulo (SP); CUT: Curitiba; POA: Porto Alegre.

Source: Instituto Brasileiro de Geografia e Estatística (Brazilian Institute of Geography and Statistics), 2023.

Project approved by the Research Ethics Committees of the institutions where interviews were conducted and the Conselho Nacional de Ética em Pesquisa (Conep - National Research Ethics Council). All interviewees digitally signed the Informed Consent Form.

### Variables and Data Sources

Data were collected through a combination of document analysis, literature review, and complementary semi-structured interviews with key actors, when necessary (Chart 1). The interviews were conducted via Google Meet, with simultaneous recording and transcription using the Tactiq app, following prior approval by the respective Research Ethics Committees (CUT, SC, DF, CE). The eight key stakeholders interviewed were physicians (3), a dentist (1), nurses (3), and an educator (1), each with at least three years of experience working in the Regulatory Complexes they represented.

**Chart 1:** Summary of data sources in each scenario, Brasília (DF), 2026.

Document title	Type	Year	Responsible entity	Source	Interviews
Study on health regulation models in the national and international context to support the revision of the guidelines of the National Policy on Regulation of the Unified Health System (SUS) – Final Report	Research report	2025	Pan American Health Organization	Internal circulation	
Ceará					
Ceará State Health Plan 2024–2027	Plan	2023	Government of the State of Ceará - State Department of Health	https://www.saude.ce.gov.br/wp-content/uploads/sites/9/2024/08/Plano_Estadual_da_Saude_2024-2027-1.pdf	Two follow-up interviews conducted in December 2023, January, and June 2024 with key stakeholders from the Regulatory Complex of the State of Ceará
State Law No. 17,195 of March 27, 2020	Law	2020	Government of the State of Ceará	https://www.esp.ce.gov.br/download/lei-estadual-n-o-17-195-de-27-de-marco-de-2020/	
Regulatory Complexes Manual	Manual	2019	Government of the State of Ceará – SES	https://www.saude.ce.gov.br/wp-content/uploads/sites/9/2019/09/MANUAL_COMPLEXO_REGULADORES_VERSAO_FINAL.pdf	
Santa Catarina					
Resolution No. 142/CIB/2016	Resolution	2016	Government of the State of Santa Catarina – SES - Bipartite Interagency Commission (CIB)	https://antigo.saude.sc.gov.br/index.php/documentos/informacoes-gerais/regulacao-1/deliberacoes-portarias/deliberacoes-regulacao-2016	A follow-up interview conducted with a key actor from the Regulatory Complex of the State of Santa Catarina in April 2024
Resolution No. 231/CIB/2016	Resolution	2016	Government of the State of Santa Catarina - SES - Bipartite Interagency Commission (CIB)	https://antigo.saude.sc.gov.br/index.php/documentos/informacoes-gerais/regulacao-1/deliberacoes-portarias/deliberacoes-regulacao-2016/12901-deliberacao-231-cib-08-12-2016-1/file	
Rio Grande do Sul					
Socioeconomic Atlas of Rio Grande do Sul	Atlas	2021	Government of the State of Rio Grande do Sul - Secretariat of Planning, Governance, and Management	https://issuu.com/spggrs/docs/atlas_socioconomico_do_rio_grande_do_sul	No supplementary interviews were conducted
CIB Resolution No. 100/07	Resolution	2007	Government of the State of Rio Grande do Sul - SES - Bipartite Interagency Commission (CIB)	https://saude.rs.gov.br/upload/arquivos/carga20170246/23104627-1340742918-cibr100-07.pdf	
CIB Resolution No. 152/09	Resolution	2009	Government of the State of Rio Grande do Sul - SES - Bipartite Interagency Commission (CIB)	https://saude.rs.gov.br/upload/arquivos/carga20170234/23103410-1340305907-cibr152-09.pdf	
CIB Resolution No. 399/2011	Resolution	2011	Government of the State of Rio Grande do Sul - SES - Bipartite Interagency Commission (CIB)	https://saude.rs.gov.br/upload/arquivos/carga20170220/23102056-1340035041-cibr399-11.pdf	
Rio Grande do Sul State Health Plan 2020–2023	Plan	2020	Government of the State of Rio Grande do Sul - SES	https://saude.rs.gov.br/upload/arquivos/202106/01164321-ma-0001-20-plano-estadual-de-saude-28-05-interativo-b.pdf	
Federal District					
Regulatory Process for Access to Healthcare (Consultations, Exams, Surgical Procedures, and Hospital Beds)	Manual	2021	Government of the Federal District - SES	https://info.saude.df.gov.br/wp-content/uploads/2022/02/Manual.Processo.GER_.GIR_05.10.2021.pdf	Four interviews conducted with key stakeholders of the CRDF in October 2023 and January 2024
2020–2023 District Health Plan	Plan	2019	Federal District Government - SES	https://www.saude.df.gov.br/plano-distrital-de-saude	
Ordinance No. 1388, dated December 12, 2018	Administrative Order	2018	Federal District Government - SES	https://www.sinj.df.gov.br/sinj/Norma/61d302d0c57548879a1302b814e804d5/Portaria_1388_12_12_2018.html	
Curitiba					
Curitiba Municipal Health Plan - Paraná 2022–2025	Plan	2021	Curitiba City Hall - SES	https://saude.curitiba.pr.gov.br/conteudo/instrumentos-de-planejamento-em-saude/1348	A follow-up interview conducted with a key actor from the Curitiba Regulatory Complex in June 2024
Health Management in Curitiba: 2027–2020	Articles	2020	Municipal Institute of Public Administration (Imap)	Volume 4, Issue 7 revista.imap.curitiba.pr.gov.br	
Porto Alegre					
Decree No. 21,904, dated March 20, 2023. Establishes the Internal Regulations of the SMS	Decree	2023	City of Porto Alegre	https://leismunicipais.com.br/a/rs/p/porto-alegre/decreto/2023/2191/21904/decreto-n-21904-2023-establishes-the-internal-regulations-of-the- -municipal-health-secretariat-sms-within-the-scope-of-the-centralized-administration-of-the-municipal-government-of-porto-alegre-pmpa-and-repeals-articles-1-through-6-of-decree-no.-15293-of-August-30-2006-and-Decree-No.-21425-of-March-23-2022	No follow-up interviews were conducted
Porto Alegre Municipal Health Plan - Rio Grande do Sul 2022–2025	Plan	2021	City of Porto Alegre – SES	https://prefeitura.poa.br/sites/default/files/usu_doc/sites/sms/PLANO%20MUNICIPAL%20DE%20SA%C3%9ADE%202022-2025.pdf	
CIB Resolution No. 228/16	Resolution	2016	Government of the State of Rio Grande do Sul - SES - Bipartite Interagency Commission (CIB)	https://saude.rs.gov.br/upload/arquivos/carga20170219/23111910-1468006415-cibr228-16.pdf	
Presentation Regulatory Complex.pptx	Presentation	2024	Procempa - Public Information and Communication Technology Company of the City of Porto Alegre	https://prefeitura.poa.br/sites/default/files/usu_doc/sites/procempa/projetos/2024/07/Apresenta%C3%A7%C3%A3o%20Complexo%20Regulador.pdf	
São Paulo					
Ordinance No. 349 of March 17, 2015	Ordinance	2015	City of São Paulo – SMS	https://legislacao.prefeitura.sp.gov.br/leis/portaria-secretaria-municipal-da-saude-349-de-10-de-abril-de-2015	No follow-up interviews were conducted
São Paulo Municipal Health Plan 2022–2025	Plan	2021	City of São Paulo – SMS	https://www.prefeitura.sp.gov.br/cidade/secretarias/upload/saude/plano_municipal_de_saude_2021_240822_versao_site.pdf	
Ordinance No. 246 of March 17, 2015	Ordinance	2015	City of São Paulo – SMS	http://legislacao.prefeitura.sp.gov.br/leis/portaria-secretaria-municipal-da-saude-246-de-18-de-marco-de-2015	
2024 Annual Health Plan	Plan	2023	City of São Paulo – SMS	https://www.prefeitura.sp.gov.br/cidade/secretarias/upload/saude/pas_2024_corrigida_2023_05_04.pdf	
Technical Instruction No. 01/2024	Technical Instruction	2024	City of São Paulo – SMS - Telehealth Planning and Monitoring Commission (CPAT)	https://prefeitura.sp.gov.br/documents/d/saude/instrutivo_teleinterconsulta_ago24-pdf	
Belo Horizonte					
Belo Horizonte Municipal Health Plan 2018–2021	Plan	2018	City of Belo Horizonte - SMS	https://prefeitura.pbh.gov.br/sites/default/files/estrutura-de-governo/saude/2025/20-05-25-smsa-pms-2018-2021.pdf	No follow-up interviews were conducted
Annual Health Plan - 2023	Plan	2022	City of Belo Horizonte – SMS	https://prefeitura.pbh.gov.br/sites/default/files/estrutura-de-governo/saude/2022/pas-2023.pdf	
Decree No. 16,882, dated April 6, 2018	Decree	2018	City of Belo Horizonte – SMS	https://prefeitura.pbh.gov.br/sites/default/files/estrutura-de-governo/saude/Decreto%2016882_2018%20Novo%20BH%20Mais%20Sau%CC%81de.pdf	
2022 Annual Management Report	Report	2022	City of Belo Horizonte – SMS	https://prefeitura.pbh.gov.br/sites/default/files/estrutura-de-governo/saude/2022/rag-2022-consolidado_formatado.pdf	
Detailed report for the previous four-month period: 1st quarter of 2023	Report	2023	City of Belo Horizonte – SMS	https://prefeitura.pbh.gov.br/sites/default/files/estrutura-de-governo/saude/2023/1_rdqa-2023_31-05-23.pdf	

SES: State Health Secretariat; SMS: Municipal Health Secretariat; CRDF: Federal District Regulatory Complex.

The variables were defined based on the domains of the Consolidated Framework for Implementation Research (CFIR), a conceptual meta-framework designed to identify factors that influence the implementation of innovations and determine their success or failure. It is organized into five main domains: intervention characteristics, external and internal context, individual characteristics, and the implementation process. For each domain, organizational, political, and operational factors related to the implementation of regulatory strategies were examined. Although the model includes several subdomains, it is recognized that its scope may not fully reflect the contexts of low- and middle-income countries. For this reason, an inductive approach was adopted, allowing for the incorporation of emerging variables and the contextual adaptation of the framework to the Brazilian scenario^17^.

### Data Analysis

Documentary analysis, literature review, and supplementary interviews enabled the description of the normative and institutional framework of the interventions, as well as the contextualization of the formulation and implementation of the innovations studied. Interview transcripts were reviewed and subjected to qualitative thematic analysis^18^ . Both techniques enabled the organization of the research *corpus* according to the CFIR domains, through discussion and consensus among the researchers, to avoid redundancies in the categorization of information within each domain.

## RESULTS

Chart 2 summarizes the patterns identified in state and municipal experiences with specialized outpatient regulation and teleconsultation. The results, organized according to the CFIR analytical domains, allowed us to identify determinants of implementation, advances, weaknesses, and unique contextual factors of the scenarios (Chart 3).

**Chart 2:** Summary of patterns observed in outpatient regulation and teleconsultation initiatives.

Domain	Patterns observed in the analyzed experiences		Source
	Outpatient Coordination	Teleconsultation	
Characteristics of the intervention	Outpatient dispatch with and without teleconsultation performed by dispatch centers or dispatch complexes, centralized in state capitals with or without decentralized structures in health regions. In large municipalities, centralized regulatory complexes predominate, with some integration with state-level coordination. Use of proprietary systems or SISREG, with varying levels of integration and interoperability; standardized clinical protocols based on national guidelines and local agreements; Implementation driven by high demand, waiting lists, inequalities in access, and the need to improve referral quality.	Centralized model (state/municipal), with variations in reach; Generally integrated with regulation; Objectives: support for PHC, qualifying referrals, reducing waiting lists, improving resolution rates, and rational use of services; Predominance of proprietary platforms; use of informal platforms in some locations; Protocols by specialty and formalized workflows; Combined synchronous and asynchronous interactions; preference for asynchronous interactions in some contexts.	Interviews with key stakeholders and document analysis
External context	Regulation supported by federal, state, and municipal regulations, with strong influence from SUS guidelines and local laws defining responsibilities; Funding is primarily state/municipal, with occasional federal support and the use of consortia/partnerships for sustainability; The pandemic suspended elective services, disrupted waiting lists, and accelerated digitization; Pressure from regulatory bodies, the media, and society for transparency, equity, and efficiency. Legal challenges regarding access were frequent.	Institutional and political support, along with external partnerships, were fundamental for regional consolidation; Diversity in governance arrangements, with institutional variations and the absence of state digital health policies; The pandemic accelerated digitization and the reorganization of care workflows; Technological advances and integration with electronic health records and digital platforms were essential for expansion and effectiveness; Hybrid financing (local and federal resources and strategic programs); Demand for effectiveness, efficiency, and specialized care drove the institutionalization of the strategy.	Interviews with key stakeholders and document analysis
Internal landscape	Limited interoperability hinders data sharing across levels of care; Training sessions take place, but they are not very systematic; CIBs are involved in agreeing on workflows and criteria; more mature models have formalized protocols and technical bodies; BI-based monitoring is adopted in some locations; transparency of waiting lists varies, with some municipalities already offering public access.	Different degrees of incorporation, institutionalization, and utilization; Governance involves coordination between managers and institutions, though with varying degrees of formalization and agreement; Training is ongoing and structured in some locations, sporadic or insufficient in others; Distance learning is well-established in Rio Grande do Sul and Porto Alegre, but less developed in other regions; Protocols are common, but clear state policies are lacking; A tool for second opinions and regulation, but without clear guidelines, it can be a barrier to specialized care.	Interviews with key stakeholders and document analysis
Characteristics of individuals	State and municipal departments coordinate regulation; Regulation Centers manage access; regional coordination offices oversee flows, protocols, and indicators; PHC professionals request referrals and enter data into the systems; Regulatory physicians evaluate, prioritize, authorize, or return requests with guidance, and support PHC via teleconsultation; Technical teams and universities provide training and ongoing support.	State and municipal health departments coordinate policies, provide infrastructure, train staff, and monitor telemedicine services; Primary care professionals record questions for technical support and to avoid unnecessary referrals; Specialist physicians respond with guidance based on scientific evidence; Managers coordinate levels of care, agree on protocols, provide training, and ensure infrastructure; Consultants also act as regulators. Telehealth centers manage systems, update protocols, and provide support and training.	Interviews with key stakeholders and document analysis
Implementation process	Strategies: creation and adaptation of clinical protocols; regionalization of access; systematic monitoring of indicators; inter-federative agreements; integration with teleconsulting and digitization of processes; Barriers: lack of full integration between systems; initial resistance; shortage of specialties and available slots; difficulties in post-referral monitoring; Facilitators: use of computerized systems; continuous training of teams; political and institutional support; use of telehealth and academic partnerships.	Strategies: gradual implementation; integration with regulatory and healthcare systems; development and use of interoperable platforms; institutional partnerships; monitoring of indicators. Facilitators: engagement and training of teams; institutional and academic support; standardized protocols and clear workflows; technological infrastructure; ongoing training. Barriers: fragmented and unintegrated systems; initial resistance from professionals; technological and connectivity limitations in some regions; shortage and turnover of professionals; lack of institutionalization in some services.	Interviews with key stakeholders and document analysis

PHC: Primary Health Care; SISREG: Regulation System; SUS: Unified Health System; BI: Business Intelligence; RS: Rio Grande do Sul.

**Chart 3:** Comparative summary of findings according to CFIR domains regarding the regulation of access to specialized care and teleconsultation in Brazilian states and municipalities.

CFIR Domain	Analytical findings	States	Municipalities
Characteristics of the intervention	Highly complex intervention, based on clinical protocols and risk classification, with teleconsultation serving as support for clinical decision-making and care coordination.	Full integration between regulation and teleconsultation in SC and RS; partial integration in CE; lack of integration in the Federal District.	Greater integration in Porto Alegre, even influencing the state of RS; partial integration in Curitiba, São Paulo, and Belo Horizonte.
External context	National SUS guidelines, judicialization, demands for transparency, and the COVID-19 pandemic as drivers of institutional change; academic partnerships as a factor of legitimacy.	Consolidated regulatory framework in SC and RS; nascent regulatory framework in CE; institutional instability in the Federal District.	Greater social and media pressure in São Paulo and Porto Alegre; response to local health emergencies in all municipal scenarios analyzed.
Internal Context	Variability in governance, interoperability of information systems, and organizational readiness; growing use of ICT and monitoring dashboards with information systems integrated with analytical intelligence capabilities to support decision-making.	High organizational maturity in SC and RS; system fragmentation and governance fragility in the Federal District; publicly accessible monitoring dashboards in the Federal District, shared with state oversight agencies.	Integration of regulation with electronic medical records in Curitiba and Belo Horizonte; public dashboards with analytical intelligence capabilities in São Paulo and Porto Alegre.
Characteristics of individuals	PHC professionals as key actors; expansion of the regulator’s role to include clinical and educational functions; professional resistance as a cross-cutting barrier.	Greater regulator–PHC integration mediated by teleconsultation in SC, RS, and CE; low professional adherence in the Federal District.	Diversification of the profile of regulators in São Paulo; greater clinical interaction in Porto Alegre.
Implementation process	Gradual implementation, prioritization of critical specialties, continuous training, gradual incorporation of ICT, and systematic monitoring of indicators.	Mature and ongoing processes in SC and RS; recent consolidation in CE; instability in the Federal District.	Consolidation of the model in Porto Alegre; incremental innovation and heterogeneous trajectories in the other municipalities.

CFIR: Consolidated Framework for Implementation Research; SC: Santa Catarina; RS: Rio Grande do Sul; CE: Ceará; DF: Federal District; SUS: Unified Health System; ICT: Information and Communication Technologies; MPDFT: Public Prosecutor’s Office of the Federal District and Territories; BI: Business Intelligence; PHC: Primary Health Care.

### (1) Characteristics of the intervention

Regulation of access to specialized care, with or without the use of teleconsultation, consists of monitoring wait times, reducing user no-shows for scheduled appointments and unnecessary procedures, coordinating the care pathway, and improving communication between teams and users^19,20^. These actions are carried out by regulation centers or regulatory complexes.

Access regulation protocols are used that establish referral criteria, case prioritization, and user pathways and flows within the RAS. Care prioritization is based on clinical severity and user vulnerabilities, regardless of the order of entry on the waiting list^21^.

Since 2008, the Ministry of Health has developed and made available to state and municipal governments the Regulation System (SISREG) as an important operational support tool. Some states and municipalities have opted to develop their own technological solutions for regulation management, often customized to address local realities and integrated with other municipal management platforms.

Teleconsultation, when integrated into referral systems, promotes interaction between PHC professionals and specialists and aims to clarify doubts regarding clinical procedures, health interventions, and issues related to the workflow^22^. It is used to support access regulation, improving clinical decision-making and reducing unnecessary patient travel. Its use, although guided by national guidelines, depends on local organization. In some settings, it is established as a mandatory step preceding referral from PHC to specialized care. In this model, the active agent initiating the process is the requesting professional themselves, who contacts the teleconsultation team.

### (2) External scenario

In all scenarios, national SUS guidelines and local legislation influenced the organization of emergency care regulation. In addition, pressure from regulatory bodies, increasing litigation, and social demands for transparency and accountability served as decisive factors in driving institutional changes. The Covid-19 pandemic had a widespread impact across all territories, leading to the suspension of elective care and longer waiting lists. The need to ensure access during the health crisis led to the reorganization of schedules, accelerated digitization, and greater adoption of telehealth tools.

Among the states, SC and RS stand out, with a regulatory framework consolidated in laws, decrees, and specific resolutions on regulation and telehealth. CE structures its actions primarily around protocols and operational initiatives of the State Telehealth Center, without a formalized regulatory framework. In municipalities, greater regulatory autonomy is observed, with local legislation and their own decrees guiding regulatory flows. POA and SPO stand out, with integrated protocols between specialized care regulation and teleconsultation.

Institutional partnerships have also proven decisive. The states of SC and RS maintain ongoing cooperation with universities and telehealth centers, which are responsible for training, evaluation, and technical-scientific support. In municipalities, academic support is equally significant, and in some cases, such as in CUT, collaboration with the Regional Medical Council (CRM) was cited as relevant to the technical legitimacy of the actions.

External factors such as institutional crises, natural disasters, and regional inequalities directly influenced the implementation of regulation in the states. In the municipalities, social and media pressure for transparency and local health emergencies, such as dengue outbreaks, stood out, requiring rapid reorganization of agendas and prioritization of cases.

### (3) Internal context

The internal organization of emergency care regulation varied across states and municipalities, reflecting different institutional arrangements, levels of technological adoption, and management capacity. In all settings, progress was observed in the hierarchization and regionalization of care pathways, as well as in criteria for access and user prioritization. The exception was the Federal District, characterized by institutional instability, fragile governance, and fragmentation of health information systems (HIS), with significant compromises to the traceability of referrals by primary care and the continuity of care.

Team training proved to be a cross-cutting element in all contexts. The states of SC and RS maintain ongoing programs and partnerships with universities. In municipalities, continuing education is also prioritized, with POA and CUT standing out for their digital platform-based training.

Integration among SIS is limited in the analyzed scenarios. SC and RS have more structured processes, with intensive use of Information and Communication Technologies (ICT) and Business Intelligence (BI) systems to monitor waiting lists and access indicators in real time. BI tools are also used in the Federal District, although integration between systems remains limited. In municipalities, SPO, POA, and CUT stand out for also promoting public access to information, with greater transparency. In BH, despite the digitization of processes, public visibility of data was still evolving.

Teleconsultation was incorporated in a heterogeneous manner. The states of RS, SC, and CE use teleconsultation platforms distinct from the regulation systems, even when they have their own telehealth platforms. The lack of interoperability between these systems was noted by CE and in the municipality of SPO. The municipality of POA also adopts separate platforms, with greater integration between the regulation and telehealth systems.

### (4) Characteristics of the individuals

The roles of actors in emergency care regulation and teleconsultation are similar. Consolidated functional structures were identified in the Dispatch Centers, which centralize activities such as managing the SIS, organizing waiting lists, validating referrals, and monitoring care provision. These structures operate through a complex network of institutional and individual actors, with continuous functions, although with local variations in the forms of execution and coordination.

In all scenarios, PHC professionals were identified as key actors in entering requests, recording clinical information, and complying with regulatory and clinical protocols. Regulating physicians perform technical mediation, assessing demands and deciding on authorization, return with guidance, denial, or referral.

In the municipality of SPO, there was an expansion of the professional profile of regulators, including nurses, physical therapists, and speech-language pathologists with different regulatory competencies. This includes technicians who support triage, document ver, and the organization of requests, suggesting greater professionalization of administrative functions.

In various contexts, teleconsultations were observed between coordinating physicians and PHC professionals to provide technical and clinical support in analyzing requests. Such integration represents an expansion of the coordinator’s role, who now acts as an access manager, clinical consultant, and educational support agent.

Institutional and academic partnerships are structural elements of the sustainability of the analyzed models, in the training of consultants, in innovation projects, in the shared management of SIS, in clinical support, and in the technical improvement of teams. Furthermore, these partnerships have enabled the continuous updating of clinical and operational protocols.

### (5) Implementation process

There are recurring strategies, advances, and weaknesses in the process of implementing regulations for access to specialized care and teleconsultation. Although each scenario has followed its own trajectory, a convergent trend toward technological modernization, institutionalization of protocols, and expansion of the educational role of telehealth has been observed.

Between 2019 and 2023, the CE restructured outpatient regulation, prioritizing critical specialties. Teleconsultation, initially focused on educational activities, became part of the regulation, incorporating both synchronous and asynchronous clinical interactions. The observed weaknesses included fragmented systems, the absence of a unified medical record, and professional resistance. The integration of teleconsultation into the regulation represented a step forward.

In SC, outpatient regulation began in 2009, with the adoption of SISREG in 2010 and the Regulation Centers in 2013. Between 2018 and 2019, the quota-based model was abandoned, and access regulation protocols with risk classification were adopted. Teleconsultation, implemented in 2007, was consolidated through training, specific protocols, an asynchronous platform, coordination with primary care, and the use of informational channels, such as messaging services. Weaknesses included initial resistance to change, technical disparities among the state’s municipalities, and a limited supply of emergency care services due to a shortage of specialists. The standardization of e protocols and the consolidation of a widespread teleconsultation system were the advances observed.

In RS, the current model for regulating access to specialized care began in 2016, inspired by the experience in POA, with gradual expansion starting in 2021. Teleconsultation was implemented in 2007 and strengthened by the TelessaúdeRS program, which, since 2012, has contributed to the standardization of protocols and integration of tools, coordinating teleconsultation, training, and the SIS. The main weaknesses were limited connectivity in some municipalities and resistance to change. Advances included the statewide expansion of the regulatory model, with the integration of telehealth.

In the Federal District, the first initiatives to regulate access to outpatient care began in 2004 with the establishment of the Appointment and Test Scheduling Coordination Center, which was consolidated in 2007 with the creation of the Regulatory Directorate. Protocols and monitoring dashboards created starting in 2024 with the support of state regulatory agencies, along with training programs, were implemented, albeit amid resistance, contractual limitations, and high turnover among managers.

Teleconsulting, initiated in 2017 through the Regula+Brasil Project, used an asynchronous model to address waiting lists; however, it was not fully integrated into outpatient regulation, as it was limited to the duration of the Project. Weaknesses were identified, such as low adherence and/or a lack of updated protocols and poor integration with primary care. The advances highlighted in the interviews included the creation of BI dashboards for decision support, shared with regulatory agencies, and greater transparency of information for the public.

In the municipalities, experiences were more varied. In CUT, regulation began in 2013 and was formalized in 2017, with strengthened clinical regulation in the basic health units and the creation of protocols with support from the Paraná Medical Council (CRM). The local SIS integrated regulation into the electronic medical record. Teleconsulting, also initiated in 2017, facilitated the regulation of access to priority specialties and the definition of care pathways. The weaknesses identified were professional resistance and a shortage of specialists, while the advances included the integration of SIS and specialty-based teleregulation.

In POA, teleconsulting, used since 2012 in partnership with TelessaúdeRS led by the university, develops and applies protocols, uses BI dashboards, and supports primary care. However, outpatient regulation gained momentum in 2015 , with the implementation of integrated proprietary SIS systems. The main weaknesses identified were: judicialization, long waiting lists, inequality in service provision, and professional resistance. Notable advances include: technological investment, technical-academic support, and the positioning of teleconsultation as a structural pillar of regulation.

In the municipality of SPO, regulation began in 2003 and was restructured in 2015 with the creation of the Municipal Coordination of Regulation, Evaluation, and Control. Local SISs were partially integrated, with regulatory protocols and telehealth initiatives. Teleconsultation began in 2017, with teledermatology in partnership with an external institution. Weaknesses included the poor integration of the SIS, professional resistance, and the lack of protocols in some areas. Advances included the professionalization of the regulation teams and the implementation of teleconsultation by care pathways.

In Belo Horizonte, outpatient triage was intensified starting in 2020 with the implementation of the local SIS, which definitively replaced SISREG in 2022, integrating with the electronic medical record and improving the traceability of care. Teleconsultation began in 2003–2004 with BH-Telessaúde and advanced with the Regula+Brasil Project (2018–2022). Investments were made in training, platform integration, and clinical triage. There are weaknesses in the formalization of protocols, monitoring, professional adherence, and the uneven incorporation of technologies. There have been advances in expanding care pathways and applying teleconsultation as a clinical screening tool.

## DISCUSSION

Although subnational governments start from common guidelines, the implementation of regulations governing access to emergency care in the SUS takes on distinct forms, determined by institutional capacity, organizational arrangements, technological innovation, and the use of telehealth. Regions that more consistently integrated clinical protocols, integrated health information systems (SIS), and teleconsultation demonstrated greater institutionalization of specialized care access regulation, better-qualified referrals, and greater potential for reducing waiting lists and wait times. Contexts marked by fragmented systems, unstable governance, and professional resistance faced greater difficulties in consolidating effective regulatory models.

The regulation of emergency care has established itself, since the 2000s, as a model that involves teams of regulators responsible for prioritizing requests originating from primary care by applying previously defined technical criteria, mediating the relationship between demand and healthcare supply through the organization of waiting lists, definition of care pathways, and continuous monitoring, to ensure orderly, equitable, and timely access to health services^6,23^ .

National policies establish guidelines for regulating access but do not define minimum parameters for operational protocols, delegating responsibility for structuring them to local managers. Although this flexibility preserves federal autonomy, it may compromise equity in access due to divergent interpretations and limit the dissemination of systematically evaluated and replicable adaptations in health policies^17^ .

The heterogeneity of regulatory processes among states and municipalities can undermine the consolidation of SUS principles. Ideally, national regulations with standardized clinical protocols guide implementation, defining margins for local adaptation without compromising effectiveness and equity. In practice, however, fragmented regional strategies prevail, with low technological integration and limited use of clinical management tools^1,17^ .

Adjustments and improvements to outpatient regulation have been occurring since 2003, with gradual advances driven by new national policies. The Covid-19 pandemic represented a disruptive milestone, accelerating the adoption of digital technologies and the reconfiguration of access regulation. Addressing this health crisis propelled telehealth as an essential tool to ensure continuity of care^11,12,15,24^ . In several countries, the pandemic led to the relaxation of regulatory standards to allow for the rapid adoption of digital tools and emergency protocols that would ensure the maintenance of services^24^. This experience also highlighted the need for advance planning and proactive policies, as formulating protocols in crisis situations can compromise the quality and consistency of care provision^25^ .

The findings of this study indicate that teleconsultation has significant potential to improve the quality of referrals, enhance the effectiveness of primary care, and reduce user wait times for specialist care, as evidenced by experiences in states such as Rio Grande do Sul, Santa Catarina, Belo Horizonte, and Recife, as reported in the literature^12,26,27^ In addition to optimizing the regulatory flow , the integration between regulation and telehealth encourages spontaneous teleconsultations, which promote coordination between PHC professionals and specialists, strengthening continuing education, interprofessional collaboration, and collaboration across levels of care^28^ .

The lack of interoperability and integration among HISs remains a weakness that hinders monitoring, evaluation, and transparency, compromising social control and evidence-based management. This challenge is shared by other countries with decentralized public systems, according to a comparative study on the integration between PHC and specialized care in Brazil and Spain^29^ .

The CFIR proved consistent for analyzing the interaction of institutional, technological, and human factors and their influence on health innovation, favoring a more structured and comparable approach across different contexts^17,30^ . There are limitations to extrapolating the results of this study to the country as a whole, although the experiences are representative of recurring patterns in policies regulating access to specialized care.

## CONCLUSION

The implementation of regulations governing access to emergency care in the SUS, analyzed using the CFIR, varied according to institutional, organizational, and technological capacities. We identified weaknesses in interoperability and integration among SIS, in the organization of specialized care provision with inequalities in care and regulatory infrastructure, in health system governance with low standardization of clinical protocols, incipient integration between PHC and specialized care, and professional resistance to change.

The regions that stood out integrated clinical and access protocols, information systems integrated with analytical intelligence resources to support regulatory processes, and strong institutionalization of regulatory guidelines. The integration of teleconsultation into regulation stood out in health system governance, particularly in improving coordination between PHC and specialized care.

The CFIR framework allowed us to understand how the interaction between contexts, actors, and processes influences the implementation of interventions, reinforcing the relevance of national guidelines capable of strengthening RAS.

## Data Availability

The entire dataset supporting the results of this study has been published in the article.
