# Supplementary material for: Access to specialized outpatiecare: an analysis of regulation in Brazilian settings
Source: Rev Saude Publica. 2026 Jun 15;60:e19. doi: 10.11606/s1518-8787.2026060007411 (PMC13271211; doi:10.11606/s1518-8787.2026060007411)
Supplement: Translation

# Translation: Acesso à atenção ambulatorial especializada: análise da regulação em cenários brasileiros

**Authors:** Thais Barros Zanette da Silva, Jozinélio Severino Teixeira, Carlos Amilcar Salgado, Luciano de Paula Camilo, Claudilene Sousa Fortaleza, Fábio Ferreira Amorim, Leila Bernarda Donato Göttems

**Affiliations:** I Universidade do Distrito Federal. Escola Superior de Ciências da Saúde. Brasília, DF, Brasil; II Universidade de Brasília. Faculdade de Ciências da Saúde. Brasília, DF, Brasil; III Organização Pan-Americana de Saúde. Brasília, DF, Brasil

**Keywords:** Sistema Único de Saúde, Regulação e Fiscalização em Saúde, Acesso aos Serviços de Saúde, Atenção Secundária à Saúde, Políticas, Planejamento e Administração em Saúde, Telemedicina

## Abstract

**OBJETIVO:**

Analisar de que maneira diferentes governos subnacionais, partindo de diretrizes nacionais comuns, desenvolveram modelos próprios de regulação do acesso à atenção especializada, com distintos graus de inovação, institucionalização e integração da telessaúde.

**MÉTODO:**

Estudo descritivo e exploratório, de abordagem qualitativa, que analisa de forma comparada a implementação da regulação do acesso à atenção especializada. Dados coletados por meio de análise documental, revisão da literatura e, em alguns casos, entrevistas com atores-chave, consolidados em descrições detalhadas de cada cenário, para composição do *corpus* da pesquisa. Análise realizada com a aplicação do *Consolidated Framework for Implementation Research* (CFIR). Foram selecionadas as experiências dos municípios de Curitiba (PR), Belo Horizonte (MG), Porto Alegre (RS) e São Paulo (SP) e dos estados do Ceará, Santa Catarina, Rio Grande do Sul e do Distrito Federal.

**RESULTADOS:**

As oito experiências brasileiras de regulação ambulatorial se caracterizam pela diversidade nos modelos organizacionais, graus de institucionalização da teleconsultoria e integração entre níveis de atenção. A adoção de tecnologias, protocolos de regulação do acesso e sistemas informatizados variou entre os locais, influenciada por fatores estruturais, normativos e contextuais. A teleconsultoria, quando integrada, qualificou os encaminhamentos e aumentou a resolutividade na atenção primária, mas sua implementação permanece desigual no território nacional.

**CONCLUSÃO:**

A implementação da regulação do acesso à atenção especializada no SUS variou conforme capacidades institucionais, organizacionais e tecnológicas. Foram identificadas fragilidades na integração entre sistemas de informação em saúde na organização e oferta da atenção especializada com desigualdades estruturais, na governança com baixa padronização de protocolos, integração limitada entre Atenção Primária à Saúde e atenção especializada e resistência profissional a mudanças. Territórios com melhores resultados integraram a teleconsultoria à regulação ambulatorial, protocolos clínicos e de acesso adotaram sistemas de informação em saúde integrados com recursos de inteligência analítica e diretrizes regulatórias institucionalizadas. O CFIR evidenciou a influência de contextos, atores e processos, reforçando a importância de diretrizes nacionais para aperfeiçoar as organizações locais.

## INTRODUÇÃO

O arranjo federativo e descentralizado do Sistema Único de Saúde (SUS) brasileiro favoreceu a ampliação do acesso à Atenção Primária à Saúde (APS), mas ainda mantém a desarticulação das Redes de Atenção à Saúde (RAS) e a desigualdade na oferta na atenção especializada (AE) entre as regiões do país^1^, especialmente em pequenos municípios, por restrições técnicas, operacionais e financeiras^2^. Entre as estratégias para o enfrentamento deste cenário, destaca-se a Política Nacional de Regulação em Saúde (PNR)^3^, que promove a cooperação intergovernamental, o compartilhamento de recursos e a integração entre os níveis de atenção. Publicada em 2008 e revisada em 2025, a PNR possui marco normativo, estrutura operacional, financiamento e sistema de informação que visa organizar fluxos assistenciais, mediar a relação entre a oferta e a demanda e promover o uso racional dos recursos disponíveis^4^. Sua implementação, contudo, revela elevada heterogeneidade no contexto brasileiro, em razão do caráter federativo do sistema, da coexistência com o setor privado e das desigualdades na capacidade institucional entre os entes federativos^5^.

No Brasil, a regulação em saúde organiza-se em três dimensões interdependentes: do acesso, da atenção e dos sistemas de saúde^6^. A regulação do acesso desempenha papel essencial na articulação entre APS e AE, por meio das centrais de regulação, protocolos e sistemas informatizados^7,8^. O conceito evoluiu nos documentos oficiais (Figura), como uma articulação de ações destinadas a ordenar e controlar o acesso à AE, com base em critérios de risco, vulnerabilidade, necessidades de saúde e disponibilidade de recursos^9,10^.

**Figura:** Infográfico com a evolução conceitual da Regulação do Acesso Ambulatorial no Brasil.

Fonte: elaborado com base nas referências 9,10,21,22.

A regulação em saúde ganhou expressiva visibilidade e fortalecimento desde meados de 2010, por meio do Programa Nacional Telessaúde Brasil Redes, ao qualificar os encaminhamentos e otimizar o uso dos serviços da AE^11^. Foram criadas novas modalidades de interação, destacando-se a teleconsultoria, definida como consulta entre profissionais de saúde para esclarecimento de dúvidas clínicas, realizada por telecomunicação bidirecional, síncrona ou assíncrona. Evidências apontam que essa prática tem sido eficaz na redução das filas de espera, ampliação da resolubilidade da APS e qualificação das decisões clínicas^14,15^. No entanto, sua implementação tem variado conforme fatores contextuais, políticos e institucionais^16^.

Este estudo tem como objetivo analisar de que maneira diferentes governos subnacionais, partindo de diretrizes nacionais comuns, desenvolveram modelos próprios de regulação do acesso à AE, com distintos graus de inovação, institucionalização e integração da telessaúde.

## MÉTODOS

### Desenho do Estudo

Estudo descritivo e exploratório, de abordagem qualitativa, que analisa de forma comparada a implementação da regulação do acesso à AE em três estados – Ceará (CE), Santa Catarina (SC) e Rio Grande do Sul (RS); quatro municípios – Belo Horizonte-MG (BH), Porto Alegre-RS (POA), São Paulo-SP (SPO), Curitiba-PR (CUT) e no Distrito Federal (DF), organizado político-administrativamente, como estado e município.

A seleção dos cenários buscou representar diferentes contextos e arranjos institucionais, com base em critérios de relevância institucional, disponibilidade de informações públicas e reconhecimento nacional, possibilitando a comparação de modelos distintos de governança e de integração entre a APS e a AE. Os cenários apresentam heterogeneidade demográfica, socioeconômica e organizacional (Tabela), compartilham a autonomia na gestão do SUS e enfrentam desafios comuns relacionados ao acesso à AE.

**Tabela:** Indicadores epidemiológicos e demográficos dos cenários selecionados.

Cenário	População (nº de habitantes)	IDH	Esperança de vida ao nascer em anos	Mortalidade infantil por 1.000 nascidos vivos	Cobertura de APS
DF	2.982.818	0,814	79,7	8,5	76,8%
SC	7.338.473	0,774	77,4	9,8	88,7%
CE	9.240.580	0,682	79,01	11,9	77,2%
RS	10.880.506	0,771	75,38	9,59	68,0%
BH	2.315.560	0,797	78,81	8,19	81,3%
SPO	11.451.245	0,783	82,62	10,23	70,6%
CUT	1.773.718	0,823	78,5	7,1	75,8%
POA	1.332.570	0,805	77,45	7,78	61,0%

IDH: Índice de Desenvolvimento Humano; APS: Atenção Primária à Saúde; DF: Distrito Federal; SC: Santa Catarina; CE: Ceará; RS: Rio Grande do Sul; BH: Belo Horizonte (MG); SPO: São Paulo (SP); CUT: Curitiba; POA: Porto Alegre.

Fonte: Instituto Brasileiro de Geografia e Estatística, 2023.

Projeto aprovado pelos Comitês de Ética em Pesquisa das instituições onde foram realizadas entrevistas e Conselho Nacional de Ética em Pesquisa. Todos os entrevistados assinaram digitalmente o Termo de Consentimento Livre e Esclarecido.

### Variáveis e Fonte de Dados

Dados coletados por meio da combinação entre análise documental, revisão de literatura e entrevistas semiestruturadas complementares com atores-chave, quando necessário (Quadro 1). As entrevistas ocorreram por meio do *Google Meet*, com gravação e transcrição simultânea pelo aplicativo *Tactiq*, após aprovação prévia dos respectivos comitês de ética (CUT, SC, DF, CE). Os oito atores-chave entrevistados eram médicos (3), dentista (1), enfermeiros (3) e pedagoga (1), com atuação mínima de três anos de atuação nos Complexos Reguladores que representavam.

**Quadro 1:** Síntese das fontes de dados em cada cenário, Brasília (DF), 2026.

Título do documento	Tipo	Ano	Entidade responsável	Fonte	Entrevistas
Estudo sobre os modelos de regulação em saúde no contexto nacional e internacional para apoiar a revisão das diretrizes da Política Nacional de Regulação do Sistema Único de Saúde (SUS) – Relatório final	Relatório de pesquisa	2025	Organização Pan-americana de Saúde	Circulação interna	
Ceará					
Plano Estadual de Saúde do Ceará 2024–2027	Plano	2023	Governo do Estado do Ceará - Secretaria de Estado de Saúde	https://www.saude.ce.gov.br/wp-content/uploads/sites/9/2024/08/Plano_Estadual_da_Saude_2024-2027-1.pdf	Duas entrevistas complementares realizadas nos meses de dezembro de 2023, janeiro e junho de 2024, com atores-chave do Complexo Regulador do Estado do Ceará
Lei Estadual nº 17.195 de 27/03/2020	Lei	2020	Governo do Estado do Ceará	https://www.esp.ce.gov.br/download/lei-estadual-n-o-17-195-de-27-de-marco-de-2020/	
Manual dos Complexos Reguladores	Manual	2019	Governo do Estado do Ceará – SES	https://www.saude.ce.gov.br/wp-content/uploads/sites/9/2019/09/MANUAL_COMPLEXO_REGULADORES_VERSAO_FINAL.pdf	
Santa Catarina					
Deliberação nº 142/CIB/2016	Deliberação	2016	Governo do Estado de Santa Catarina – SES - Comissão Intergestores Bipartite (CIB)	https://antigo.saude.sc.gov.br/index.php/documentos/informacoes-gerais/regulacao-1/deliberacoes-portarias/deliberacoes-regulacao-2016	Uma entrevista complementar realizada com ator chave do Complexo Regulador do Estado de SC em abril de 2024
Deliberação nº 231/CIB/2016	Deliberação	2016	Governo do Estado de Santa Catarina - SES - Comissão Intergestores Bipartite (CIB)	https://antigo.saude.sc.gov.br/index.php/documentos/informacoes-gerais/regulacao-1/deliberacoes-portarias/deliberacoes-regulacao-2016/12901-deliberacao-231-cib-08-12-2016-1/file	
Rio Grande do Sul					
Atlas socioeconômico do Rio Grande do Sul	Atlas	2021	Governo do Estado do Rio Grande do Sul - Secretaria de Planejamento, Governança e Gestão	https://issuu.com/spggrs/docs/atlas_socioconomico_do_rio_grande_do_sul	Não foram realizadas entrevistas complementares
Resolução CIB nº 100/07	Resolução	2007	Governo do Estado do Rio Grande do Sul - SES - Comissão Intergestores Bipartite (CIB)	https://saude.rs.gov.br/upload/arquivos/carga20170246/23104627-1340742918-cibr100-07.pdf	
Resolução CIB nº 152/09	Resolução	2009	Governo do Estado do Rio Grande do Sul - SES - Comissão Intergestores Bipartite (CIB)	https://saude.rs.gov.br/upload/arquivos/carga20170234/23103410-1340305907-cibr152-09.pdf	
Resolução CIB nº 399/2011	Resolução	2011	Governo do Estado do Rio Grande do Sul - SES - Comissão Intergestores Bipartite (CIB)	https://saude.rs.gov.br/upload/arquivos/carga20170220/23102056-1340035041-cibr399-11.pdf	
Plano Estadual de Saúde do Rio Grande do Sul 2020–2023	Plano	2020	Governo do Estado do Rio Grande do Sul - SES	https://saude.rs.gov.br/upload/arquivos/202106/01164321-ma-0001-20-plano-estadual-de-saude-28-05-interativo-b.pdf	
Distrito Federal					
Processo Regulatório de Acesso à Assistência (Consultas, Exames, Procedimentos Cirúrgicos e Leitos)	Manual	2021	Governo do Distrito Federal - SES	https://info.saude.df.gov.br/wp-content/uploads/2022/02/Manual.Processo.GER_.GIR_05.10.2021.pdf	Quatro entrevistas realizadas com atores-chave do CRDF em outubro de 2023 e janeiro de 2024
Plano Distrital de Saúde 2020–2023	Plano	2019	Governo do Distrito Federal - SES	https://www.saude.df.gov.br/plano-distrital-de-saude	
Portaria nº 1388, de 12 de dezembro de 2018	Portaria	2018	Governo do Distrito Fedeal - SES	https://www.sinj.df.gov.br/sinj/Norma/61d302d0c57548879a1302b814e804d5/Portaria_1388_12_12_2018.html	
Curitiba					
Plano Municipal de Saúde de Curitiba - Paraná 2022–2025	Plano	2021	Prefeitura de Curitiba - SES	https://saude.curitiba.pr.gov.br/conteudo/instrumentos-de-planejamento-em-saude/1348	Uma entrevista complementar realizada com ator chave do Complexo Regulador de Curitiba em junho de 2024
A Gestão da Saúde em Curitiba: gestão 2027 a 2020	Artigos	2020	Instituto Municipal e Administração Pública (Imap)	Volume 4, número 7 revista.imap.curitiba.pr.gov.br	
Porto Alegre					
Decreto nº 21.904, de 20 de março de 2023. Estabelece o Regimento Interno da SMS	Decreto	2023	Prefeitura de POA	https://leismunicipais.com.br/a/rs/p/porto-alegre/decreto/2023/2191/21904/decreto-n-21904-2023-estabelece-o-regimento-interno-da-secretaria-municipal-de-saude-sms-no-ambito-da-administracao-centralizada-da-prefeitura-municipal-de-porto-alegre-pmpa-e-revoga-os-arts-1-a-6-do-decreto-n-15293-de-30-de-agosto-de-2006-e-o-decreto-n-21425-de-23-de-marco-de-2022	Não foram realizadas entrevistas complementares
Plano Municipal de Saúde de Porto Alegre - Rio Grande do Sul 2022–2025	Plano	2021	Prefeitura de POA – SES	https://prefeitura.poa.br/sites/default/files/usu_doc/sites/sms/PLANO%20MUNICIPAL%20DE%20SA%C3%9ADE%202022-2025.pdf	
Resolução CIB nº 228/16	Resolução	2016	Governo do Estado do Rio Grande do Sul - SES - Comissão Intergestores Bipartite (CIB)	https://saude.rs.gov.br/upload/arquivos/carga20170219/23111910-1468006415-cibr228-16.pdf	
Apresentação Complexo Regulador.pptx	Apresentação	2024	Procempa - Empresa Pública de Tecnologia da Informação e Comunicação da Prefeitura de Porto Alegre	https://prefeitura.poa.br/sites/default/files/usu_doc/sites/procempa/projetos/2024/07/Apresenta%C3%A7%C3%A3o%20Complexo%20Regulador.pdf	
São Paulo					
Portaria nº 349 de 17 de março de 2015	Portaria	2015	Prefeitura de São Paulo – SMS	https://legislacao.prefeitura.sp.gov.br/leis/portaria-secretaria-municipal-da-saude-349-de-10-de-abril-de-2015	Não foram realizadas entrevistas complementares
Plano Municipal de Saúde de São Paulo 2022–2025	Plano	2021	Prefeitura de São Paulo – SMS	https://www.prefeitura.sp.gov.br/cidade/secretarias/upload/saude/plano_municipal_de_saude_2021_240822_versao_site.pdf	
Portaria nº 246 de 17 de março de 2015	Portaria	2015	Prefeitura de São Paulo – SMS	http://legislacao.prefeitura.sp.gov.br/leis/portaria-secretaria-municipal-da-saude-246-de-18-de-marco-de-2015	
Programação anual de saúde 2024	Plano	2023	Prefeitura de São Paulo – SMS	https://www.prefeitura.sp.gov.br/cidade/secretarias/upload/saude/pas_2024_corrigida_2023_05_04.pdf	
Instrutivo técnico nº 01/2024	Instrutivo técnico	2024	Prefeitura de São Paulo – SMS - Comissão de Planejamento e Acompanhamento da Telessaúde (CPAT)	https://prefeitura.sp.gov.br/documents/d/saude/instrutivo_teleinterconsulta_ago24-pdf	
Belo Horizonte					
Plano Municipal de Saúde de Belo Horizonte 2018–2021	Plano	2018	Prefeitura de Belo Horizonte - SMS	https://prefeitura.pbh.gov.br/sites/default/files/estrutura-de-governo/saude/2025/20-05-25-smsa-pms-2018-2021.pdf	Não foram realizadas entrevistas complementares
Programação Anual de Saúde - 2023	Plano	2022	Prefeitura de Belo Horizonte – SMS	https://prefeitura.pbh.gov.br/sites/default/files/estrutura-de-governo/saude/2022/pas-2023.pdf	
Decreto nº 16.882, de 6 de abril de 2018	Decreto	2018	Prefeitura de Belo Horizonte – SMS	https://prefeitura.pbh.gov.br/sites/default/files/estrutura-de-governo/saude/Decreto%2016882_2018%20Novo%20BH%20Mais%20Sau%CC%81de.pdf	
Relatório anual de gestão 2022	Relatório	2022	Prefeitura de Belo Horizonte – SMS	https://prefeitura.pbh.gov.br/sites/default/files/estrutura-de-governo/saude/2022/rag-2022-consolidado_formatado.pdf	
Relatório detalhado do quadrimestre anterior 1° quadrimestre de 2023	Relatório	2023	Prefeitura de Belo Horizonte – SMS	https://prefeitura.pbh.gov.br/sites/default/files/estrutura-de-governo/saude/2023/1_rdqa-2023_31-05-23.pdf	

SES: Secretaria Estadual de Saúde; SMS: Secretaria Municipal de Saúde; CRDF: Complexo Regulador do Distrito Federal.

As variáveis foram definidas a partir dos domínios do *Consolidated Framework for Implementation Research* (CFIR), metaestrutura conceitual para identificar fatores que influenciam a implementação de inovações e determinam seu sucesso ou fracasso. Organiza-se em cinco domínios principais: características da intervenção, cenário externo e interno, características dos indivíduos e processo de implementação. Para cada domínio, foram observados fatores organizacionais, políticos e operacionais da implementação das estratégias de regulação. Embora o modelo inclua diversos subdomínios, reconhece-se que seu escopo pode não refletir integralmente os contextos de países de baixa e média renda. Por essa razão, foi adotada uma abordagem indutiva, permitindo a incorporação de variáveis emergentes e a adequação contextual do referencial ao cenário brasileiro^17^.

### Análise de Dados

A análise documental, da literatura e as entrevistas complementares propiciaram a descrição do arcabouço normativo e institucional das intervenções, a contextualização da formulação e da implementação das inovações estudadas. As transcrições das entrevistas foram revisadas e submetidas à análise temática qualitativa^18^. Ambas as técnicas possibilitaram organizar o *corpus* da pesquisa de acordo com os domínios do CFIR, por meio de discussão e consenso entre os pesquisadores, para evitar redundâncias na categorização das informações dentro de cada domínio.

## RESULTADOS

O Quadro 2 sintetiza os padrões identificados nas experiências estaduais e municipais de regulação ambulatorial especializada e de teleconsultoria. Os resultados, organizados conforme os domínios analíticos do CFIR, permitiram identificar fatores determinantes da implementação, os avanços, as fragilidades e fatores contextuais singulares dos cenários (Quadro 3).

**Quadro 2:** Síntese dos padrões observados nas experiências de regulação ambulatorial e teleconsultoria.

Domínio	Padrões observados nas experiências analisadas		Fonte
	Regulação ambulatorial	Teleconsultoria	
Características da intervenção	Regulação ambulatorial com e sem teleconsultoria realizada pelas centrais de regulação ou complexos reguladores, centralizados nas capitais dos estados com ou sem estruturas descentralizadas em regiões de saúde. Nos grandes municípios predominam complexos reguladores centralizados com alguma integração com a regulação do estado. Uso de sistemas próprios ou SISREG, com níveis distintos de integração e interoperabilidade; protocolos clínicos padronizados, baseados em diretrizes nacionais e pactuações locais; Implantação motivada por alta demanda, filas, desigualdades no acesso e necessidade de qualificar encaminhamentos.	Modelo centralizado (estadual/ municipal), com variações na capilaridade; Em geral, integrada à regulação; Objetivos: suporte à APS, qualificação de encaminhamentos, redução de filas, maior resolutividade e uso racional da oferta; Predomínio de plataformas próprias; uso de plataformas informais em alguns locais; Protocolos por especialidade e fluxos formalizados; Interações síncronas e assíncronas combinadas; preferência por interações assíncronas em alguns contextos.	Entrevistas com atores-chave e análise documental
Cenário externo	Regulação respaldada por normativas federais, estaduais e municipais, com forte influência das diretrizes do SUS e leis locais que definem competências; Financiamento majoritariamente estadual/municipal, com apoio federal pontual e uso de consórcios/parcerias para sustentabilidade; Pandemia suspendeu serviços eletivos, desorganizou filas e acelerou a digitalização; Pressão de órgãos de controle, mídia e sociedade por transparência, equidade e agilidade. A judicialização do acesso foi recorrente.	Apoio institucional, político e parcerias externas foram fundamentais para consolidação regional; Diversidade nos arranjos de governança, com variações institucionais e ausência de políticas estaduais de saúde digital; Pandemia acelerou a digitalização e reorganização dos fluxos assistenciais; Avanço tecnológico e integração com prontuário eletrônico e plataformas digitais foram essenciais para expansão e efetividade; Financiamento híbrido (recursos locais, federais e programas estratégicos); Demanda por resolutividade, eficiência e atenção especializada impulsionou a institucionalização da estratégia.	Entrevistas com atores-chave e análise documental
Cenário interno	Interoperabilidade limitada dificulta o compartilhamento de dados entre níveis de atenção; Capacitações ocorrem, mas são pouco sistematizadas; CIB atuam na pactuação de fluxos e critérios; modelos mais maduros têm protocolos formalizados e instâncias técnicas; Monitoramento por BI é adotado em alguns locais; a transparência das filas varia, com alguns municípios já oferecendo acesso público.	Diferentes graus de incorporação, institucionalização e utilização; Governança envolve articulação gestor e instituição, porém com formalização e pactuação diversas; Capacitação é contínua e estruturada em alguns locais, pontual ou insuficiente em outros; Teleducação forte no RS e Porto Alegre, menos desenvolvida em outras regiões; Protocolos são comuns, mas faltam políticas estaduais claras; Ferramenta para segunda opinião e regulação, mas sem diretrizes claras pode ser barreira ao acesso especializado.	Entrevistas com atores-chave e análise documental
Características dos indivíduos	Secretarias estaduais e municipais coordenam a regulação; Centrais de Regulação gerenciam o acesso; coordenadorias regionais supervisionam fluxos, protocolos e indicadores; Profissionais da APS solicitam encaminhamentos e registram dados nos sistemas; Médicos reguladores avaliam, priorizam, autorizam ou devolvem solicitações com orientações, e apoiam a APS via teleconsultoria; Equipes técnicas e universidades oferecem capacitação e suporte contínuo.	Secretarias (estaduais/municipais) coordenam políticas, garantem infraestrutura, capacitam e monitoram a teleconsultoria; Profissionais da APS registram dúvidas para suporte técnico e evitar encaminhamentos desnecessários; Médicos especialistas respondem com orientações baseadas em evidências científicas; Gestores articulam níveis de atenção, pactuam protocolos, capacitam e garantem infraestrutura; Consultores atuam também como reguladores. Núcleos de telessaúde gerenciam sistemas, atualizam protocolos, oferecem suporte e capacitação.	Entrevistas com atores-chave e análise documental
Processo de implementação	Estratégias: criação e adaptação de protocolos clínicos; regionalização do acesso; monitoramento sistemático de indicadores; pactuações interfederativas; integração com a teleconsultoria e digitalização dos processos; Barreiras: falta de integração plena entre sistemas; resistência inicial; escassez de especialidades e vagas; dificuldades no monitoramento pós encaminhamento; Facilitadores: uso de sistemas informatizados; capacitação contínua das equipes; apoio político-institucional; uso da telessaúde e parcerias acadêmicas.	Estratégias: implantação gradual; integração com regulação e sistemas assistenciais; desenvolvimento e uso de plataformas interoperáveis; parcerias institucionais; monitoramento de indicadores. Facilitadores: engajamento e qualificação das equipes; apoio institucional e acadêmico; protocolos padronizados e fluxos claros; infraestrutura tecnológica; capacitação contínua. Barreiras: sistemas fragmentados e sem integração; resistência inicial dos profissionais; limitações tecnológicas e de conectividade em algumas regiões; escassez e rotatividade de profissionais; falta de institucionalização em alguns serviços.	Entrevistas com atores-chave e análise documental

APS: Atenção Primária à Saúde; SISREG: Sistema de Regulação; SUS: Sistema Único de Saúde; BI: *Business Intelligence*; RS: Rio Grande do Sul.

**Quadro 3:** Síntese comparativa dos achados segundo os domínios do CFIR na regulação do acesso à AE e na teleconsultoria em estados e municípios brasileiros.

Domínio CFIR	Achados analíticos	Estados	Municípios
Características da intervenção	Intervenção de elevada complexidade, baseada em protocolos clínicos e classificação de risco, com a teleconsultoria atuando como apoio à decisão clínica e à coordenação do cuidado.	Integração plena entre regulação e teleconsultoria em SC e RS; integração parcial no CE; ausência de integração no DF.	Maior integração em Porto Alegre, inclusive influenciando o estado do RS; integração parcial em Curitiba, São Paulo e Belo Horizonte.
Cenário externo	Diretrizes nacionais do SUS, judicialização, demandas por transparência e pandemia de covid-19 como indutores de mudança institucional; parcerias acadêmicas como fator de legitimidade.	Arcabouço normativo consolidado em SC e RS; marco regulatório incipiente no CE; instabilidade institucional no DF.	Maior pressão social e midiática em São Paulo e Porto Alegre; resposta a emergências sanitárias locais em todos os cenários municipais analisados.
Cenário interno	Variabilidade na governança, interoperabilidade dos sistemas de informação e prontidão organizacional; uso crescente de TIC e painéis de monitoramento com sistemas de informação integrados com recursos de inteligência analítica para suporte à tomada de decisão.	Alta maturidade organizacional em SC e RS; fragmentação dos sistemas e fragilidade de governança no DF; painéis de monitoramento de acesso público no DF, compartilhados com órgãos de controle estatal.	Integração da regulação ao prontuário eletrônico em Curitiba e Belo Horizonte; painéis públicos com recursos de inteligência analítica em São Paulo e Porto Alegre.
Características dos indivíduos	Profissionais da APS como atores-chave; ampliação do papel do regulador para funções clínicas e educativas; resistência profissional como barreira transversal.	Maior integração regulador–APS mediada pela teleconsultoria em SC, RS e CE; baixa adesão profissional no DF.	Diversificação do perfil dos reguladores em São Paulo; maior interação clínica em Porto Alegre.
Processo de implementação	Implantação gradual, priorização de especialidades críticas, capacitação contínua, incorporação gradual de TIC e monitoramento sistemático dos indicadores.	Processos maduros e contínuos em SC e RS; consolidação recente no CE; instabilidade no DF.	Consolidação do modelo em Porto Alegre; inovação incremental e trajetórias heterogêneas nos demais municípios.

CFIR: *Consolidated Framework for Implementation Research*; AE: atenção especializada; SC: Santa Catarina; RS: Rio Grande do Sul; CE: Ceará; DF: Distrito Federal; SUS: Sistema Único de Saúde; TIC: Tecnologias de Informação e Comunicação; MPDFT: Ministério Público do Distrito Federal e Territórios; BI: *Business Intelligence*; APS: Atenção Primária à Saúde.

### (1) Características da intervenção

A regulação do acesso à AE, com ou sem o uso da teleconsultoria, consiste na realização de atividades de monitoramento dos tempos de espera, redução do absenteísmo dos usuários aos atendimentos agendados e de realização de procedimentos desnecessários, coordenação do percurso assistencial e aprimoramento da comunicação entre equipes e usuários^19,20^. Essas ações são realizadas pelas centrais de regulação ou complexos reguladores.

Utilizam-se protocolos de regulação do acesso que estabelecem os critérios de encaminhamento, de priorização de casos e os itinerários e fluxos dos usuários na RAS. A priorização dos atendimentos ocorre com base na gravidade clínica e nas vulnerabilidades do usuário, independentemente da ordem de entrada na lista de espera^21^.

O Ministério da Saúde desenvolveu e disponibilizou aos entes federativos desde 2008, o Sistema de Regulação (SISREG) como uma importante ferramenta de apoio operacional. Alguns estados e municípios optaram pelo desenvolvimento de soluções tecnológicas próprias para a gestão da regulação, frequentemente customizadas para atender às realidades locais e integradas a outras plataformas de gestão municipal.

A teleconsultoria, quando integrada aos complexos reguladores, promove a interação entre profissionais da APS e especialistas e objetiva elucidar dúvidas sobre procedimentos clínicos, ações de saúde e questões relativas ao processo de trabalho^22^. É utilizada para apoiar a regulação do acesso, qualificando a decisão clínica e reduzindo deslocamentos desnecessários de pacientes. Seu uso, embora orientado por diretrizes nacionais, depende da organização local. Em algumas experiências, é estabelecida como etapa obrigatória precedente ao encaminhamento da APS para a AE. Nesse modelo, o agente ativo que inicia o processo é o próprio profissional solicitante, que aciona a equipe de teleconsultoria.

### (2) Cenário externo

Em todos os cenários, as diretrizes nacionais do SUS e as legislações locais exerceram influência sobre a organização da regulação da AE. Além disso, pressões de órgãos de controle, judicialização crescente e demandas sociais por transparência e responsividade, atuaram como vetores decisivos na indução de mudanças institucionais. A pandemia de covid-19 teve um impacto transversal em todos os territórios, provocando a suspensão de atendimentos eletivos e o aumento das filas de espera. A necessidade de garantir o acesso durante a crise sanitária levou à reorganização das agendas, à informatização acelerada e à maior adesão às ferramentas de telessaúde.

Entre os estados, destacam-se SC e RS, com arcabouço normativo consolidado em leis, decretos e resoluções específicas sobre a regulação e telessaúde. O CE estrutura suas ações principalmente em protocolos e iniciativas operacionais do Núcleo Estadual de Telessaúde, sem um marco regulatório formalizado. Nos municípios, observa-se maior autonomia normativa, com legislações locais e decretos próprios que orientam os fluxos de regulação. Destacam-se POA e SPO, com protocolos integrados entre regulação da AE e teleconsultoria.

As parcerias institucionais também se mostraram decisivas. Os estados de SC e RS mantêm cooperação contínua com universidades e centros de telessaúde, responsáveis por atividades de capacitação, avaliação e apoio técnico-científico. Nos municípios, o apoio acadêmico é igualmente expressivo, e em alguns casos, como em CUT, a colaboração com o Conselho Regional de Medicina (CRM), foi apontada como relevante para a legitimidade técnica das ações.

Fatores externos como crises institucionais, desastres naturais e desigualdades regionais influenciaram diretamente a implementação da regulação nos estados. Nos municípios, sobressaíram-se a pressão social-midiática por transparência e as emergências sanitárias locais, tais como os surtos de dengue, que exigiram reorganização rápida das agendas e priorização de casos.

### (3) Cenário interno

A organização interna da regulação da AE variou entre estados e municípios, refletindo diferentes arranjos institucionais, níveis de incorporação tecnológica e capacidade de gestão. Em todos os cenários identificou-se evolução na hierarquização e regionalização dos fluxos assistenciais, critérios de acesso e de priorização de usuários. Exceto o DF, marcado por instabilidade institucional, governança frágil e fragmentação dos sistemas de informação em saúde (SIS), com forte comprometimento da rastreabilidade dos encaminhamentos pela APS e da continuidade do cuidado.

A capacitação das equipes mostrou-se um elemento transversal em todos os contextos. Os estados de SC e RS mantêm programas contínuos e parcerias com universidades. Nos municípios, a educação permanente também é priorizada, com destaque para POA e CUT com qualificações por plataformas digitais.

A integração entre os SIS é limitada nos cenários analisados. SC e RS apresentam processos mais estruturados, com uso intensivo de Tecnologias de Informação e Comunicação e sistemas de *Business Intelligence* (BI) no monitoramento das listas de espera e indicadores de acesso em tempo real. Ferramentas de BI também são utilizadas no DF, embora ainda com limitações de integração entre sistemas. Nos municípios, SPO, POA e CUT se sobressaem por também favorecerem acesso público a informações, com maior transparência. Em BH, apesar da informatização dos processos, a visibilidade pública dos dados ainda estava em evolução.

A teleconsultoria foi incorporada de forma heterogênea. Os estados RS, SC e CE utilizam plataformas de teleconsultoria distintas dos sistemas de regulação, mesmo quando há plataformas próprias de telessaúde. A falta de interoperabilidade entre esses sistemas foi apontada pelo CE e no município de SPO. O município de POA também adota plataformas separadas, com maior integração entre os sistemas de regulação e telessaúde.

### (4) Características dos indivíduos

As funções dos atores da regulação da AE e da teleconsultoria são similares. Identificou-se estruturas funcionais consolidadas nas Centrais de Regulação, que concentram as atividades de gestão dos SIS, organização das listas de espera, validação de encaminhamentos e monitoramento da oferta assistencial. Essas estruturas operam por uma rede complexa de atores institucionais e individuais, com funções contínuas, ainda que com variações locais nas formas de execução e de articulação.

Em todos os cenários, os profissionais da APS foram identificados como atores-chave na inserção das solicitações, no registro das informações clínicas e no cumprimento dos protocolos de regulação e clínicos. Os médicos reguladores realizam a mediação técnica, avaliando as demandas e decidindo pela autorização, devolução com orientação, negação ou reencaminhamento.

No município de SPO, verificou-se ampliação do perfil profissional dos reguladores, incluindo enfermeiros, fisioterapeutas e fonoaudiólogos com diferentes competências regulatórias. Incluem-se técnicos que apoiam as atividades de triagem, verificação documental e organização das solicitações, sugerindo maior profissionalização das funções administrativas.

Em diversos contextos, observou-se a realização de teleconsultorias entre médicos reguladores e profissionais da APS, para o apoio técnico e clínico na análise das demandas. Tal integração representa uma ampliação no papel do regulador, que passa a atuar como gestor do acesso, consultor clínico e agente de apoio educacional.

As parcerias institucionais e acadêmicas são elementos estruturantes de sustentabilidade dos modelos analisados, na formação de consultores, em projetos de inovação, na gestão compartilhada dos SIS, no suporte clínico e no aperfeiçoamento técnico das equipes. Além disso, essas parcerias têm possibilitado a atualização contínua dos protocolos clínicos e operacionais.

### (5) Processo de implementação

Existem estratégias, avanços e fragilidades recorrentes no processo de implementação da regulação do acesso a AE e da teleconsultoria. Embora cada cenário tenha seguido trajetórias próprias, observou-se um movimento convergente de modernização tecnológica, institucionalização dos protocolos e ampliação do papel educativo da telessaúde.

Entre 2019 e 2023, o CE reestruturou a regulação ambulatorial, priorizando especialidades críticas. A teleconsultoria, inicialmente voltada a ações educativas, passou a integrar a regulação, com interações clínicas síncronas e assíncronas. As fragilidades observadas foram os sistemas fragmentados, ausência de prontuário unificado e resistência profissional. A integração da teleconsultoria à regulação representou um avanço.

Em SC, a regulação ambulatorial começou em 2009, com adoção do SISREG em 2010 e das Centrais de Regulação em 2013. Entre 2018 e 2019, abandonou-se o modelo por cotas, adotando-se protocolos de regulação do acesso com classificação de risco. A teleconsultoria, implantada em 2007, consolidou-se com capacitação, protocolos específicos, plataforma assíncrona e articulação com APS e uso de canais informacionais, como os serviços de mensagens. As fragilidades incluíram resistência inicial às mudanças, desigualdade técnica entre os municípios do estado, oferta limitada de serviços de AE pela escassez de especialistas. A padronização dos protocolos e consolidação de uma teleconsultoria capilarizada foram os avanços observados.

No RS, o modelo vigente de regulação do acesso à AE começou em 2016, inspirada na experiência de POA, com expansão gradual a partir de 2021. A teleconsultoria foi implantada em 2007 e fortalecida pelo programa TelessaúdeRS, que, desde 2012, contribuiu na padronização de protocolos e integração de ferramentas, articulando teleconsultoria, capacitações e SIS. As principais fragilidades foram conectividade limitada em alguns municípios e resistência à mudança. Os avanços incluíram a expansão estadual do modelo regulatório, com integração da telessaúde.

No DF, a regulação do acesso à AE teve suas primeiras iniciativas em 2004, com o estabelecimento da Central de Regulação de Marcação de Consultas e Exames, consolidada em 2007, com a criação da Diretoria de Regulação. Protocolos, painéis de monitoramento criados a partir de 2024 com o apoio de órgãos de controle do Estado e capacitações foram implantados, embora com resistências, limitações contratuais e alta rotatividade de gestores.

A teleconsultoria, iniciada em 2017 por meio do Projeto Regula+Brasil, usou um modelo assíncrono para qualificação das listas de espera, porém não foi integrada plenamente à regulação ambulatorial, restrita à vigência do Projeto. Identificaram-se fragilidades como baixa adesão e/ou falta de protocolos atualizados e frágil integração com a APS. Os avanços apontados nas entrevistas foram a criação de painéis com BI para apoio a decisão, compartilhados com órgãos de controle e maior transparência de informações para a sociedade.

Nos municípios, as experiências foram mais heterogêneas. Em CUT, a regulação começou em 2013 e foi oficializada em 2017, com fortalecimento da regulação clínica nas unidades básicas de saúde e criação de protocolos com apoio do CRM do Paraná. O SIS local integrou a regulação ao prontuário eletrônico. A teleconsultoria, também iniciada em 2017, favoreceu a regulação do acesso a especialidades prioritárias e a definição de fluxos assistenciais. As fragilidades apontadas foram a resistência profissional e escassez de especialistas e os avanços foram a integração dos SIS e a telerregulação por especialidades.

Em POA, a teleconsultoria, utilizada desde 2012 em parceria com o TelessaúdeRS liderado pela universidade, desenvolve e aplica protocolos, usa painéis de BI e suporte à APS. Entretanto, a regulação ambulatorial ganhou força em 2015, com a implantação de SIS próprios integrados. As principais fragilidades identificadas foram: a judicialização, as longas filas, a desigualdade na oferta e a resistência profissional. Nos avanços, destacam-se: o investimento tecnológico, o apoio técnico-acadêmico e o posicionamento da teleconsultoria como estruturante da regulação.

No município de SPO, a regulação começou em 2003 e foi reestruturada em 2015 com a criação da Coordenação Municipal de Regulação, Avaliação e Controle. Os SIS locais foram parcialmente integrados, com protocolos de regulação e iniciativas de telessaúde. A teleconsultoria iniciou em 2017, com teledermatologia em parceria com instituição externa. As fragilidades incluíram a integração precária dos SIS, a resistência profissional e a falta de protocolos em algumas áreas. Os avanços foram a profissionalização das equipes de regulação e a implantação de teleconsultoria por linhas de cuidado.

Em BH, a regulação ambulatorial foi intensificada a partir de 2020 com a implantação do SIS local, que substituiu o SISREG definitivamente em 2022, integrando-se ao prontuário eletrônico e melhorando a rastreabilidade do cuidado. A teleconsultoria iniciou em 2003–2004 com o BH-Telessaúde e avançou com o Projeto Regula+Brasil (2018–2022). Investiu-se em capacitação, integração de plataformas e triagem clínica. Há fragilidades na formalização de protocolos, monitoramento, adesão dos profissionais e incorporação desigual de tecnologias. Há avanços na ampliação das linhas de cuidado e aplicação da teleconsultoria como triagem clínica.

## DISCUSSÃO

Embora os governos subnacionais partam de diretrizes comuns, a implementação da regulação do acesso à AE no SUS assume configurações distintas, determinadas pela capacidade institucional, arranjos organizacionais, inovação tecnológica e uso da telessaúde. Os territórios que integraram de forma mais consistente protocolos clínicos, SIS integrados e teleconsultoria apresentaram maior institucionalização da regulação do acesso a AE, melhor qualificação dos encaminhamentos e maior potencial de redução das listas e tempos de espera. Contextos marcados por sistemas fragmentados, governança instável e resistência profissional enfrentaram maiores dificuldades para consolidar modelos efetivos de regulação.

A regulação da AE consolidou-se, a partir dos anos 2000, como um modelo que prevê equipes de reguladores responsáveis por priorizar solicitações oriundas da APS aplicando critérios técnicos previamente definidos, mediando a relação entre a demanda e a oferta assistencial por meio da organização de listas de espera, definição de fluxos e monitoramento contínuo, para garantir um acesso ordenado, equitativo e oportuno aos serviços de saúde^6,23^.

As políticas nacionais estabelecem diretrizes para a regulação do acesso, mas não definem parâmetros mínimos para os protocolos operacionais, delegando aos gestores locais a responsabilidade de estruturá-los. Embora essa flexibilidade preserve a autonomia federativa, pode comprometer a equidade no acesso, por interpretações divergentes e limitar a disseminação de adaptações sistematicamente avaliadas e replicáveis nas políticas de saúde^17^.

A heterogeneidade dos processos de regulação entre estados e municípios pode fragilizar a consolidação dos princípios do SUS. Idealmente, uma regulamentação nacional com protocolos clínicos padronizados orienta a implementação, definindo margens de adaptação local sem prejuízo da efetividade e da equidade. Na prática, contudo, prevalecem estratégias regionais fragmentadas, com baixa integração tecnológica e pouca utilização de ferramentas de gestão clínica^1,17^.

Os ajustes e os aprimoramentos da regulação ambulatorial vêm ocorrendo desde 2003, com avanços graduais impulsionados por novas políticas nacionais. A pandemia de covid-19 representou um marco disruptivo, acelerando a adoção de tecnologias digitais e a reconfiguração da regulação do acesso. O enfrentamento dessa crise sanitária impulsionou a telessaúde como instrumento essencial para garantir a continuidade dos cuidados^11,12,15,24^. Em diversos países, a pandemia levou à flexibilização de normas regulatórias para permitir a rápida adoção de ferramentas digitais e protocolos emergenciais que assegurassem a manutenção dos serviços^24^. Essa experiência também evidenciou a necessidade de planejamento prévio e políticas de antecipação, pois formular protocolos em situações de crise pode comprometer a qualidade e a consistência da oferta assistencial^25^.

Os achados deste estudo apontam que a teleconsultoria tem alto potencial de impacto sobre a qualificação dos encaminhamentos, na resolutividade da APS e a redução do tempo de espera dos usuários para a AE, conforme experiências como as do RS, SC, BH e Recife publicadas na literatura^12,26,27^ Além de otimizar o fluxo regulatório, a integração entre regulação e telessaúde estimula teleconsultorias espontâneas, que promovem a articulação entre profissionais da APS e especialistas, fortalecendo a educação permanente, a colaboração interprofissional e entre níveis de atenção^28^.

A falta de interoperabilidade e integração entre os SIS permanece como uma fragilidade que dificulta o monitoramento, a avaliação e a transparência, comprometendo o controle social e a gestão baseada em evidências. Esse desafio é compartilhado por outros países com sistemas públicos descentralizados, segundo estudo comparativo sobre a integração entre APS e AE no Brasil e na Espanha^29^.

O CFIR mostrou-se consistente para a análise da interação dos fatores institucionais, tecnológicos e humanos e sua influência na inovação em saúde, favorecendo uma abordagem mais estruturada e comparável entre diferentes contextos^17,30^. Há limitações na extrapolação dos resultados deste estudo para o país, embora as experiências sejam representativas de padrões recorrentes nas políticas de regulação do acesso à AE.

## CONCLUSÃO

A implementação da regulação do acesso à AE no SUS, analisada com a aplicação do CFIR, variou segundo as capacidades institucionais, organizacionais e tecnológicas. Identificaram-se fragilidades na interoperabilidade e integração entre os SIS, na oferta organização da AE com desigualdades na infraestrutura assistencial e de regulação, na governança do sistema de saúde com baixa padronização de protocolos clínicos, integração incipiente entre APS e AE e resistência profissional a mudanças.

Os territórios que se destacaram integraram protocolos clínicos e de acesso, sistemas de informação integrados com recursos de inteligência analítica para suporte aos processos regulatórios e forte institucionalização das diretrizes de regulação. A integração da teleconsultoria à regulação destacou-se na governança do sistema de saúde, sobretudo na melhoria da articulação entre APS e AE.

O CFIR permitiu compreender como a interação entre contextos, atores e processos influencia a implementação de intervenções, reforçando a relevância de diretrizes nacionais capazes de fortalecer as RAS.

## Data Availability

Todo o conjunto de dados que dá suporte aos resultados deste estudo foi publicado no artigo.
